# Ocular Blood Flow Measurements in Healthy White Subjects Using Laser Speckle Flowgraphy

**DOI:** 10.1371/journal.pone.0168190

**Published:** 2016-12-13

**Authors:** Nikolaus Luft, Piotr A. Wozniak, Gerold C. Aschinger, Klemens Fondi, Ahmed M. Bata, René M. Werkmeister, Doreen Schmidl, Katarzyna J. Witkowska, Matthias Bolz, Gerhard Garhöfer, Leopold Schmetterer

**Affiliations:** 1 Department of Clinical Pharmacology, Medical University of Vienna, Vienna, Austria; 2 Department of Ophthalmology, Kepler University Hospital, Linz, Austria; 3 University Eye Hospital, Ludwig Maximilians University, Munich, Germany; 4 Department of Ophthalmology, Medical University of Warsaw, Warsaw, Poland; 5 Center for Medical Physics and Biomedical Engineering, Medical University of Vienna, Vienna, Austria; 6 Institute of Applied Physics, Vienna University of Technology, Vienna, Austria; 7 Singapore Eye Research Institute, Singapore; 8 Lee Kong Chian School of Medicine, Nanyang Technological University, Singapore; University of Sydney, AUSTRALIA

## Abstract

**Purpose:**

To assess the feasibility and reliability of Laser Speckle Flowgraphy (LSFG) to measure ocular perfusion in a sample of healthy white subjects and to elucidate the age-dependence of the parameters obtained.

**Methods:**

This cross-sectional study included 80 eyes of 80 healthy, non-smoking white subjects of Western European descent between 19 and 79 years of age. A commercial LSFG instrument was applied to measure ocular blood flow at the optic nerve head (ONH) three successive times before and after pharmacological pupil dilation. The mean blur rate (MBR), a measure of relative blood flow velocity, was obtained for different regions of the ONH. Eight parameters of ocular perfusion derived from the pulse-waveform analysis of MBR including blowout time (BOT) and falling rate (FR) were also recorded.

**Results:**

Artifact-free LSFG images meeting the quality criteria for automated image analysis were obtainable in 93.8% without pupil dilation and in 98.8% with pharmacological pupil dilation. Measurements of MBR showed excellent repeatability with intraclass correlation coefficients ≥ 0.937 and were barely affected by pupil dilation. The majority of pulse-waveform derived variables exhibited equally high repeatability. MBR-related blood flow indices exhibited significant age dependence (p<0.001). FR (r = 0.747, p<0.001) and BOT (r = -0.714, p<0.001) most strongly correlated with age.

**Conclusions:**

LSFG represents a reliable method for the quantitative assessment of ocular blood flow in white subjects. Our data affirms that the LSFG-derived variables FR and BOT may be useful biomarkers for age-related changes in ocular perfusion.

## Introduction

Some of the most prevalent eye diseases including glaucoma, age-related macular degeneration and diabetic retinopathy are associated with abnormalities in ocular perfusion.[1,2] As of today, there is no gold-standard method for the measurement of ocular blood flow available. For retinal circulation, absolute measurements of blood velocity have been realized by bidirectional Laser Doppler Velocimetry (LDV), a technique that utilizes the optical Doppler effect (i.e. the frequency shift of laser light backscattered by moving erythrocytes).[3,4] Absolute measurements of blood flow can be obtained by combining LDV with measurements of the diameter of the retinal vessel under study, as for example with the fundus-camera based Dynamic Vessel Analyzer (DVA).[5] Doppler Fourier-domain Optical Coherence Tomography (D-OCT) represents a relatively novel approach that exploits the optical Doppler effect with high-resolution spectral-domain OCT technology.[6–9] D-OCT overcomes some of the technical limitations inherent to LDV, as it allows for precise depth resolution of flow and the 3-dimensional visualization of the perfused vessel of the posterior pole. As D-OCT can also precisely determine the vessel lumen, only one instrument is required for the absolute measurement of both blood velocity and flow. Both LDV and D-OCT, however require a high amount of examiner skill and patient compliance and, hence, are not available for the use in clinical practice.

Measuring optic nerve head (ONH) blood flow is technically more challenging because of the complex vascular supply of this anatomical region. Laser Doppler Flowmetry (LDF) has been used for quantifying ONH perfusion [2,10], a method that also operates based on the optical Doppler shift of light scattered by moving red blood cells. LDF gives relative readings of blood velocity, blood volume and blood flow and is particularly suitable for the assessment of temporal changes in ONH perfusion (e.g. during changes in ocular perfusion pressure, OPP).[10] However, no instrument is commercially available.

In recent years, Laser Speckle Flowgraphy (LSFG) has rapidly arisen as a promising technique for the two-dimensional assessment of ocular blood flow in humans. LSFG enables non-invasive, patient- and examiner-friendly quantitative estimation of perfusion at the ONH, retina and choroid, simultaneously, in less than 5 seconds. Validation of the technique for the measurement of ONH perfusion has been established by means of direct comparison with the hydrogen gas clearance method [11,12] and the microsphere method.[13] LSFG has helped add to the understanding of perfusion status and its role in the pathophysiology of numerous ocular diseases.[13–24] For example, LSFG has substantiated the hypothesis that ONH perfusion continuously decreases with the progression of glaucomatous disease. [13,14] Furthermore, LSFG has ben employed in a study that correlated systemic oxidative stress with decreased ONH perfusion suggesting that both factors may play a role in the pathogenesis of normal tension glaucoma.[15] Moreover, it has revealed that early normal tension glaucoma patients exhibit a significantly altered capillary ONH perfusion profile as compared with healthy controls.[22] As a further example, LSFG has helped corroborate the hypothesis that choroidal hyperperfusion may be involved in the development of central serous chorioretinopathy.[19,21]

LSFG utilizes a laser probe with a relatively long wavelength of 830nm, thus enabling blood flow recordings from choroidal tissue. The intensity of the LSFG signal originating from the choroid is to a large degree determined by the level of pigment contained in the retinal pigment epithelium. White subjects are known to exhibit lower levels of fundus pigmentation as compared with people of Asian descent. Hence, the use of LSFG in Whites may be hampered by a possible oversaturation of the LSFG image arising from the hyper-intense choroidal signal. As of today, LSFG has gained widespread use in Japan, however, no data on LSFG measurements in white subjects exists.

In the present study, we set out to assess the feasibility and reliability of LSFG to measure ocular perfusion in a sample of healthy white subjects. In addition, we aimed to elucidate the necessity and influence of pharmacological pupil dilation in LSFG as well as the age-dependence of the obtained parameters of ocular microcirculation. This study succeeded in achieving its predefined aims as feasibility, reliability and independency from pupil dilation of the measurement modality could be demonstrated.

## Methods

### Subjects

This cross-sectional study included 80 healthy white subjects of European descent that were selected by the Department of Clinical Pharmacology of the Medical University of Vienna. All research adhered to the guidelines set forth in the Declaration of Helsinki and this specific study was approved by the Ethics Committee of our institution (Ethikkommission der Medizinischen Universität Wien; approval number EK1342/2015). After explanation of the nature and possible consequences of the study, written informed consent was obtained from all subjects.

Within the two weeks prior to the actual study day, all subjects underwent a comprehensive screening examination that comprised medical history, physical examination, best-corrected visual acuity testing using standard Early Treatment of Diabetic Retinopathy Study (ETDRS) charts, slit-lamp examination including indirect funduscopy, measurement of intraocular pressure (IOP) using Goldmann applanation tonometry, measurements of systolic blood pressure (SBP) and diastolic blood pressure (DBP) with automated oscillometry and a urine pregnancy test in women with childbearing potential. Exclusion criteria were smoking, ametropia ≥ 6 diopters, contact lens wear, any ocular surgery within 3 months prior to participation in the study, significant opacities of the optical media (e.g. corneal scars, LOCS-II grading ≥ 3, posterior capsule opacification or vitreous opacities), any other relevant ocular disease or abnormality, any clinically relevant illness as judged by the investigators, uncontrolled hypertension with systolic blood pressure (SBP) ≥160mmHg and/or diastolic blood pressure (DBP) ≥ 100mmHg, current or planned pregnancy or lactation as well as a blood donation in the three weeks prior to the study. If both eyes of a subject were eligible, the study eye was randomly selected. Subjects were instructed to abstain from alcohol and stimulating beverages containing xanthine derivatives (tea, coffee, cola-like drinks) in the 12 hours preceding the study as it has been shown that these substances may alter ocular perfusion.[25,26] Mean arterial blood pressure (MAP), pulse pressure amplitude (PPA) and ocular perfusion pressure (OPP) for the seated position were calculated as follows: MAP = SBP + 1/3 (SBP–DBP); PPA = SBP–DBP; OPP = 2/3 MAP–IOP.

### Protocol

On the study day, subjects were scheduled to arrive at the study site between 7:30 a.m. and 4.00 p.m. After a 10-minute sitting period in a quiet and air-conditioned room with the temperature maintained at 22–23°C, measurements of ocular blood flow were performed using Laser Speckle Flowgraphy (LSFG). At first, three consecutive LSFG scans were acquired with a natural scotopic pupil. Thereafter, pharmacological dilation of the pupil was achieved by topical instillation of 0.5% tropicamide eye drops (Mydriaticum Agepha Augentropfen; Agepha Ges.m.b.H., Vienna, Austria). After a further 20-minute resting period, a second set of three consecutive LSFG measurements was acquired. All scans were acquired in near darkness under identical ambient conditions by one of two experienced examiners (N.L. and P.A.W.). To capture a LSFG scan, the subject was seated comfortably and the head was positioned against the forehead bar and chin rest. Before each measurement, the subject was encouraged to blink, then, to hold his or her eyes open and refrain from blinking during scanning. The subject was asked to lean back and the chinrest was readjusted between scans. Finally, measurements of intraocular pressure (IOP) and systemic hemodynamics (SBP, DBP) were performed.

### Laser Speckle Flowgraphy

**Fig 1:**
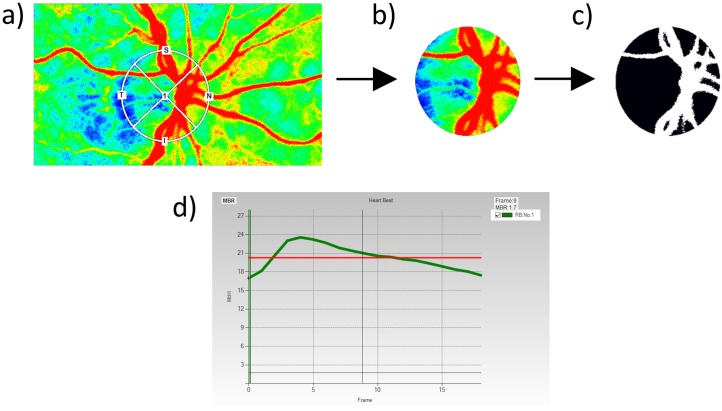
Laser Speckle Flowgraphy (LSFG) scan of the optic nerve head (ONH) area. (a) The ONH margin is to be delineated using an ellipsoid region of interest with variable size and radii. (b) The selected area representing the entire optic disc is taken into account for further analysis using the “vessel extraction” function. (c) The “vessel extraction” function distinguishes between areas of visible surface vessels (white) and ONH tissue areas (black). (d) Typical pulse-waveform curve (green) with a steep incline during the systolic phase and a flatter decline during the diastolic phase. The red line indicates the mean level of mean blur rate (MBR).

In the present study, a commercially available LSFG system (LSFG-NAVI; Softcare Co., Ltd., Fukuoka, Japan) was used to measure ocular blood flow at the ONH. The principles of LSFG have been previously described in detail.[27–31] In short, when a rough surface is illuminated with a coherent light source (e.g. a laser) the backscattered light gives the appearance of a consistent scatter pattern (i.e. the speckle pattern). Moving particles (e.g. corpuscular blood components) within the field of view cause a distinct variation in the speckle pattern in the form of a decrease in the speckle contrast and the speckle variation. After acquisition of the speckle pattern with a digital camera, these fluctuations in the speckle pattern can be analyzed in order to generate flow information. The LSFG device used in the present study consists of a fundus camera equipped with a diode laser with a wavelength of 830 nm and a digital charge-coupled device camera (750 x 360 pixels). The primary output parameter of LSFG, mean blur rate (MBR), constitutes a measure of relative blood flow velocity and is expressed in arbitrary units (AU). A total of 118 images are continuously acquired at a rate of 30 frames per second with an exposure time of 1/500 seconds over a time period of approximately 4 seconds. Accompanying analysis software (LSFG Analyzer, Version 3.1.58; Softcare Co., Ltd.) automatically detected the beginning and the end of the cardiac cycles recorded within the 4 seconds acquisition time. Images corresponding to the identical phases of the cardiac cycle were normalized to one image sequence depicting a complete cardiac cycle. The average signal intensity within each of the 750 x 360 pixels over the entire cardiac cycle was calculated in order to render the so called “composite map” depicting the distribution of mean blood flow during one cardiac cycle in the ocular fundus (Fig 1a). In this color-coded map, the ONH area is to be manually delineated by positioning an ellipsoid region of interest at the ONH margin using the image analysis software. In the present study, identical position and size of the band was maintained in all subsequent scans of the same subject using the “follow up scan” function of the software. After image acquisition, vessel and tissue areas within the ONH area were automatically detected by the software using the so-called “vessel extraction” function (Fig 1b and 1c). Thereby, a threshold for MBR signal intensity is automatically calculated by using digital cross-section analysis in order to discriminate between visible surface vessels and ONH tissue areas. Thus, MBR can be either determined for the total ONH area (referred to as MA, “mean MBR of all area”) or, separately, for vessel (MV, “mean MBR of vascular area”) and tissue areas (MT, “mean MBR of tissue area”).

**Fig 2:**
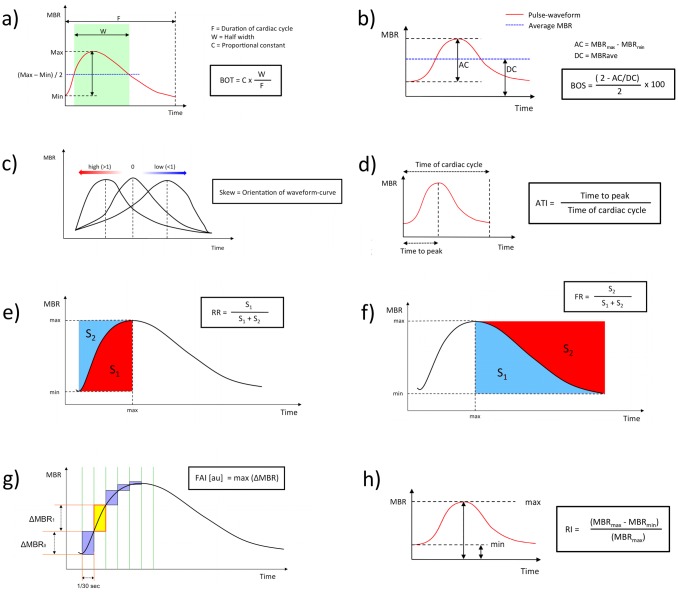
Calculation of the pulse-waveform parameters. (a) Blowout time (BOT) is defined as the ratio of the half width (i.e. the time that the waveform is higher than half of the mean of the minimum and maximum signal) to the duration of one complete cardiac cycle. High BOT is an indicator of well-maintained perfusion in between two heartbeats. (b) Similarly, blowout score (BOS) is considered as an index of the blood flow that is maintained between heartbeats and is calculated from the difference of the maximum and the minimum MBR as well as the average MBR. High BOS indicates a high constancy of blood flow during the cardiac cycle. (c) Skew serves as a measure of the asymmetry of the waveform distribution. It was developed as an indicator of the condition of the systemic circulatory system. A skew value of 0 describes a perfectly symmetrical waveform shape. Skew is positive if the distribution is leftward and negative if the distribution is rightward. As the blood flow in arteries rises more quickly than in veins, arterial skew is greater than venous skew. Skew also increases with a steeper decline of the waveform curve after the peak indicating a more rapid drop-off in blood flow after the peak. (d) Acceleration time index (ATI) is defined as the ratio of time before the pulse-wave peak value is reached to the duration of the entire heartbeat. (e) The indices rising rate (RR) and (f) falling rate (FR) characterize the steepness of the ascending and, respectively, descending portion of the waveform curve. Higher values indicate a more sudden increase, or decrease, of MBR. (g) The flow acceleration index (FAI) represents the highest increment in MBR between two frames. (h) The resistivity index (RI) is the ratio of the difference between maximum and minimum MBR to the maximum MBR.

In addition, the LSFG Analyzer software provides numerous parameters characterizing the shape of the MBR waveform during one cardiac cycle (“pulse-waveform analysis”) for assessment of the dynamics of ocular blood flow.[32–34] These additional parameters can be separately calculated for MV (corresponding to the large vessels within the ONH area) or for MT (corresponding to the ONH microvasculature). Definitions and equations for the calculation of these pulse-waveform parameters is provided in detail in Fig 2. In the present study, all pulse-waveform analyses were based on the pulse waveform obtained for the ONH microcirculation (MT).

### Statistical analysis

Descriptive data are presented as mean and standard deviation. A p-value of <0.05 was used as an indicator of statistical significance. All statistical analysis was done with SPSS Statistics (Version 22.0.0.1 for Mac; IBM).

At first, histogram frequency analysis and the Shapiro-Wilk test was performed to confirm the normal distribution of data. The Chi square test was applied to test the null hypothesis that there is no difference in the success rate of LSFG scans (calculated as the ratio of the number of successful scans and the number of all scans) before and after pharmacological pupil dilation. An LSFG scan was classified as successful if the scan could be obtained without any artifacts (e.g. due to the pupil margin constraining the field of view) and the captured image passed the quality check performed automatically by the analysis software. Measurement repeatability was calculated based on the coefficient of variation (COV; in %) defined as the ratio of the mean standard deviation of repeated measurements to the overall mean. In addition, the intraclass correlation coefficient (ICC) for the three consecutive measurements was calculated using a repeated-measures ANOVA model. Pearson coefficients were calculated for the correlations of subject age and PPA with LSFG-derived perfusion indices. Linear regression analysis was applied to test the null hypothesis that LSFG-derived parameters do not exhibit a linear correlation with age and, furthermore, with PPA. Paired samples t-test was used to test the null hypothesis that LSFG-derived parameters obtained before and after pupil dilation are comparable. Independent samples t-tests were applied to test the null hypothesis that no differences exist in the ocular, systemic and LSFG-derived parameters between male and female participants. For the analysis of age- and sex-related differences, we used the mean value of three successive LSFG measurements performed in mydriasis.

## Results

### Subjects

A total of 40 male and 40 female white subjects were included equally distributed among four age groups (group 1: 18–34 years, group 2: 35–49 years, group 3: 50–64 years, group 4: 65–80 years). Each group consisted of 10 male and 10 female subjects. A summary of participants’ baseline characteristics is provided in Table 1. Histogram frequency analysis and the Shapiro-Wilk test confirmed the normal distribution of the analyzed LSFG-derived parameters irrespective of age group, sex and pupil dilation (data not shown).

**Table 1:** Demographic and baseline characteristics of subjects.

Parameter	Total	Age Group 1	Age Group 2	Age Group 3	Age Group 4	Male	Female	t-Test
	∑ = 80	(18–34 years; n = 20)	(35–49 years; n = 20)	(50–64 years; n = 20)	(65–80 years; n = 20)	n = 40	n = 40	(p-value)
Age (years)	48.9 ± 17.4	25.6 ± 4.6	43.4 ± 3.9	55.3 ± 4.5	71.4 ± 4.5	48.8 ± 16.9	49.0 ± 18.1	0.95
BMI (kg/m2)	25.8 ± 4.2	23.0 ± 3.6	27.3 ± 3.7	26.2 ± 4.6	26.9 ± 3.6	26.4 ± 4.0	25.3 ± 4.4	0.22
MRSE (diopters)	-0.3 ± 1.6	-0.9 ± 1.3	-0.6 ± 1.3	-0.6 ± 1.7	0.9 ± 1.3	-0.3 ± 1.7	-0.2 ± 1.5	0.72
LOCS (grade)	0.6 ± 0.7	0 ± 0	0.3 ± 0.5	1.0 ± 0.47*	1.3 ± 0.7*	0.7 ± 0.8*	0.6 ± 0.6*	0.53
SBP (mmHg)	128 ± 13	124 ± 10	125 ± 11	127 ± 15	136 ± 12	132.2 ± 10.9	123.5 ± 13.6	0.002
DBP (mmHg)	81 ± 9	77 ± 8	81 ± 9	82 ± 10	83 ± 8	82.6 ± 8.2	78.5 ± 9.1	0.04
HR (bpm)	70 ± 10	74 ± 10	73 ± 10	68 ± 11	67 ± 6	68.3 ± 9.0	72.4 ± 10.5	0.06
MAP (mmHg)	96 ± 9	92 ± 8	95 ± 9	97 ± 11	101 ± 8	99.1 ± 8.0	93.5 ± 9.7	0.006
IOP (mmHg)	12.8 ± 2.4	12.7 ± 2.2	13.2 ± 2.8	12.1 ± 1.9	13.3 ± 2.7	13.1 ± 2.4	12.5 ± 2.4	0.31
OPP (mmHg)	51 ± 6	49 ± 5	50 ± 7	53 ± 7	54 ± 5	53.0 ± 5.6	49.8 ± 6.7	0.02
PPA (mmHg)	47 ± 10	47 ± 8	45 ± 8	45 ± 10	52 ± 12	50 ± 10	45 ± 10	0.04

All data are presented as mean ± standard deviation. The independent samples t-test (p-values provided in last column) was performed to test the hypothesis that no differences exist in the respective demographic/baseline parameters provided in the first column between the male (n = 40) and the female (n = 40) subgroup. SD, Standard Deviation; BMI, Body Mass Index; MRSE, Manifest Refraction Spherical Equivalent; LOCS, Lens Opacities Classification System; SBP, Systolic Blood Bressure; DBP, Diastolic Blood Pressure; HR, Heart Rate; bmp, Bearts Per Minute; MAP, Mean Arterial Pressure; IOP, Intraocular Pressure; OPP, Ocular Perfusion Pressure; PPA, Pulse Pressure Amplitude;

* One (n = 1) pseudophakic eye excluded.

### Measurement success rate

Without pupil dilation, a total of 11 LSFG scans of four subjects were not possible due to small pupil size. A further four scans of three subjects with reduced fixation compliance were excluded because the captured images did not meet the quality criteria for automated image analysis. In mydriasis, all 240 scans of the 80 subjects were successfully obtained. However, in three subjects, one scan had to be excluded for insufficient image quality. Hence, pupil dilation increased the success rate of LSFG measurements from 93.8% (225/240) to 98.8% (237/240) with p = 0.004.

### Repeatability

Table 2 summarizes the calculated repeatability indices, COV and ICC, for all LSFG-derived parameters obtained with and without pupil dilation, respectively. Favorable indices of repeatability were observed for all MBR-related parameters (MA, MV and MT) with COVs of 6.81% or lower and ICCs of 0.937 or higher. For the majority of pulse-waveform parameters, a comparably high level of repeatability was detected. BOT, BOS, FR, FAI and RI showed ICCs of ≥0.9 and skew and ATI showed ICCs of ≥0.85. RR was the only parameter with an ICC lower than 0.85. Pharmacological pupil dilation did not change the accuracy of LSFG measurements (Table 2).

**Table 2:** Repeatability Indices for LSFG-derived parameters.

LSFG Parameter	Before Pupil Dilation		After Pupil Dilation	
	COV (%)	ICC	COV (%)	ICC
**MA**	4.77	0.984	4.70	0.981
**MV**	6.81	0.937	6.44	0.942
**MT**	6.11	0.974	5.72	0.976
**BOT**	5.30	0.914	5.62	0.907
**BOS**	1.86	0.970	2.62	0.953
**Skew**	9.23	0.888	9.90	0.876
**ATI**	7.00	0.886	7.88	0.853
**RR**	5.57	0.748	5.57	0.730
**FR**	4.52	0.873	4.18	0.901
**FAI**	11.19	0.945	9.62	0.966
**RI**	4.96	0.966	6.33	0.943

LSFG (Laser Speckle Flowgraphy), COV (Coefficient of Variation), ICC (Intraclass Correlation Coefficient), MA (Mean Blur Rate of All Optic Nerve Head Area), MV (Mean Blur Rate of Vascular Area), MT (Mean Blur Rate of Tissue Area), BOT (Blowout Time), BOS (Blowout Score), ATI (Acceleration Time Index), RR (Rising Rate), FR (Falling Rate), FAI (Flow Acceleration Index), RI (Resistivity Index)

### Effect of pupil dilation

Paired t-test revealed no effect of pharmacological pupil dilation on the MBR-related values MA, MV and MT (Table 3). In contrast, pupil dilation appeared to affect all pulse-waveform analysis-derived parameters (all with p≤0.01). BOT, BOS and ATI decreased and skew, RR, FR, FAI and RI (Table 3) showed an increase after pupil dilation.

**Table 3:** LSFG parameters obtained before and after pupil dilation.

LSFG Parameter	Before Pupil Dilation	After Pupil Dilation	t-Test
	(mean ± SD)	(mean ± SD)	(p-value)
**MA**	24.5 ± 5.3	24.3 ± 5.0	0.32
**MV**	43.0 ± 6.9	42.6 ± 6.6	0.24
**MT**	12.8 ± 2.8	12.8 ± 2.8	0.63
**BOT**	49.9 ± 5.2	49.0 ± 5.0	<0.01
**BOS**	75.7 ± 5.0	74.1 ± 5.3	<0.01
**Skew**	12.4 ± 1.9	13.0 ± 1.9	<0.01
**ATI**	29.3 ± 3.6	28.6 ± 3.4	<0.01
**RR**	12.2 ± 0.8	12.4 ± 0.8	<0.01
**FR**	13.2 ± 1.0	13.4 ± 1.0	<0.01
**FAI**	1.75 ± 0.47	1.82 ± 0.55	0.01
**RI**	0.37 ± 0.06	0.39 ± 0.06	<0.01

LSFG (Laser Speckle Flowgraphy), SD (Standard Deviation), MA (Mean Blur Rate of All Optic Nerve Head Area), MV (Mean Blur Rate of Vascular Area), MT (Mean Blur Rate of Tissue Area), BOT (Blowout Time), BOS (Blowout Score), ATI (Acceleration Time Index), RR (Rising Rate), FR (Falling Rate), FAI (Flow Acceleration Index), RI (Resistivity Index)

### Age dependence

**Fig 3:**
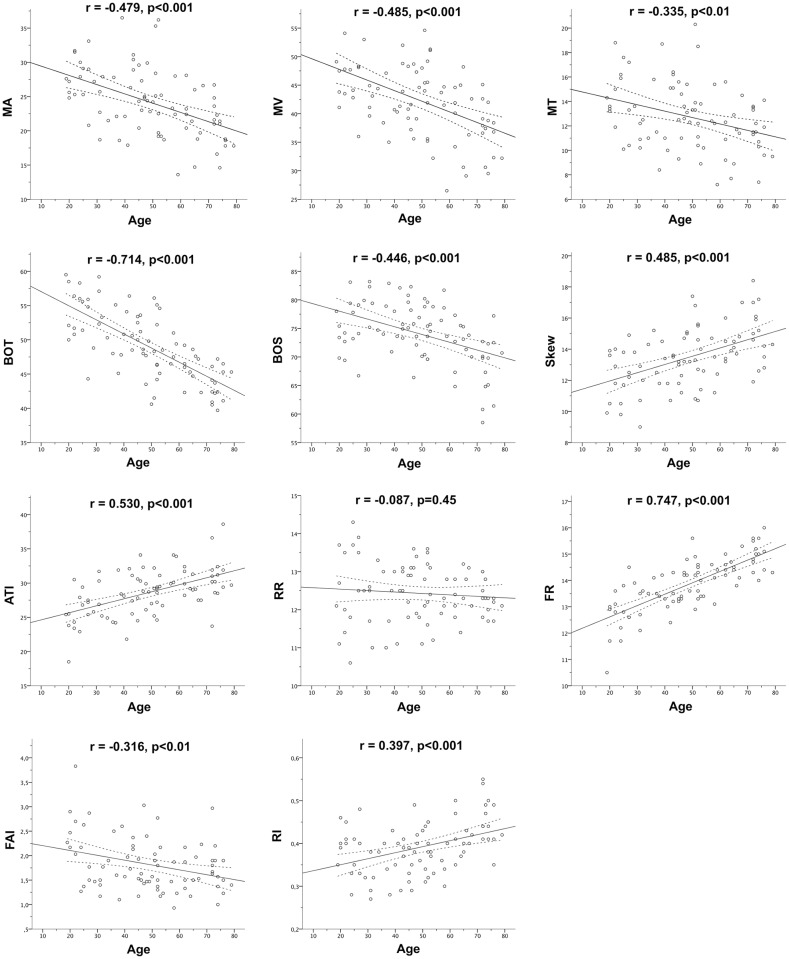
Age dependence of LSFG-derived parameters. Correlations between Laser Speckle Flowgraphy (LSFG)-derived parameters and age. Dashed lines indicate the 95% confidence intervals of the linear regression line (solid). Pearson’s r as well as the computed p-value is given for each correlation.

As depicted in Fig 3, all three MBR-related indices (MA, r = -0.479, p<0.001; MV, r = -0.485, p<0.001; MT, r = -0.335; p<0.01) significantly decreased with age. Moreover, all pulse-waveform parameters with the exception of RR (p = 0.45) showed significant age dependence and most were correlated with PPA (Table 4). The indices FR (r = 0.747, p<0.001) and BOT (r = -0.714, p<0.001) most strongly correlated with age. Generally, the correlations between LSFG-derived indices and PPA were weaker than the respective correlations with age.

**Table 4:** Age dependence of LSFG-derived parameters and their association with pulse pressure amplitude.

LSFG Parameter	Correlation with Age		Correlation with PPA	
	Pearson's r	p-value	Pearson's r	p-value
**MA**	-0.479	<0.001	-0.302	0.01
**MV**	-0.485	<0.001	-0.150	0.19
**MT**	-0.335	0.002	-0.212	0.06
**BOT**	-0.714	<0.001	-0.256	0.02
**BOS**	-0.446	<0.001	-0.421	<0.001
**Skew**	0.485	<0.001	0.252	0.02
**ATI**	0.530	<0.001	-0.206	0.07
**RR**	-0.087	0.45	-0.151	0.18
**FR**	0.747	<0.001	0.222	0.048
**FAI**	-0.316	<0.001	-0.007	0.95
**RI**	0.397	<0.001	0.393	<0.001

LSFG (Laser Speckle Flowgraphy), PPA (Pulse Pressure Amplitude), MA (Mean Blur Rate of All Optic Nerve Head Area), MV (Mean Blur Rate of Vascular Area), MT (Mean Blur Rate of Tissue Area), BOT (Blowout Time), BOS (Blowout Score), ATI (Acceleration Time Index), RR (Rising Rate), FR (Falling Rate), FAI (Flow Acceleration Index), RI (Resistivity Index)

### Sex dependence

**Fig 4:**
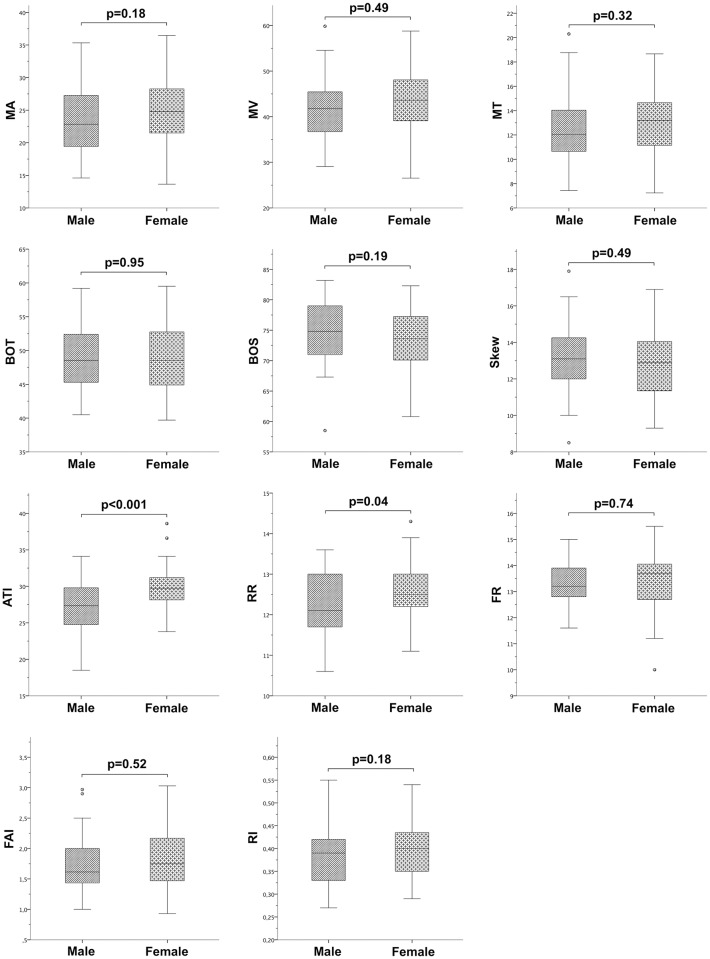
Sex dependence of LSFG-derived parameters. Boxplots comparing Laser Speckle Flowgraphy (LSFG)-derived parameters between male and female subjects.

As given in Table 1, statistically significant differences in the ocular and systemic parameters were observed for SBP, DBP, MAP, OPP and PPA, which were higher in the male than in the female subgroup. Fig 4 shows the boxplots created for LSFG-derived parameters obtained for male and female subjects. A sex-related difference was only detected for the pulse-waveform analysis-derived parameters ATI (29.9 ±2.9 versus 27.3 ±3.4; p<0.001) and RR (12.6 ±0.7 versus 12.3 ±0.8; p = 0.04), which were both higher in females than in males.

## Discussion

The primary purpose of this study was to assess the feasibility and reproducibility of Laser Speckle Flowgraphy (LSFG) as a method for quantification of ocular blood flow in white subjects. To our knowledge, the present work is the first to apply this non-invasive technique in a sample representative of a healthy white population.

Herein, we demonstrated the feasibility of LSFG to quantify ocular blood flow in the ONH area in white subjects. The values obtained for the MBR-related parameters MA, MV and MT [32–40] as well as the readings of pulse-waveform analysis derived parameters (e.g. BOT, Skew, FR) [22,32,34,40,41] were in concordance with data previously published for healthy Japanese populations. The success rate of LSFG scans was satisfactory (93.8%) with natural (scotopic) pupils, however, pharmacological dilation of the pupil expanded the success rate, which allowed for at least one successful measurement in all subjects. The primary output parameter of LSFG is mean blur rate (MBR), which can be calculated separately for the entire ONH area (MA), the vascular (MV) and the tissue area of the ONH (MT). In the present study, we demonstrated excellent repeatability for readings of MA, MV and MT at the ONH with ICCs ranging between 0.94 and 0.98. Values for ICC can range from 0 to 1, the latter representing perfect repeatability. A value above 0.9 indicates acceptable clinical repeatability.[42,43] Our findings are in accordance with previous studies exclusively including Japanese subjects.[39,44] In addition, we demonstrated that MBR-related variables could be obtained with the same favorable level of repeatability irrespective of pharmacological pupil dilation.

Furthermore, in this work we analyzed the repeatability of a plethora of pulse-waveform parameters. ICC values above 0.9 were detected for the majority of the parameters analyzed regardless of pupil dilation. As the only index found with an ICC of <0.85, rising rate (RR) exhibited lowest repeatability. Previously, only Shiga et al.[22] had assessed the repeatability of blowout time (BOT), skew and acceleration time index (ATI) in a mixed sample of Japanese subjects with normal tension glaucoma and healthy controls. In their study, a “good” level of repeatability was reported for these variables with coefficients of variation (COVs) ranging from 5.4% to 8.7%. Unfortunately, no other indices of repeatability, which are adjusted for the effects of the scale of measurements (e.g. ICCs), were provided.

In the vast majority of clinical studies using LSFG, all measurements were performed after pharmacological pupil dilation.[32,36,38,40,44–48] While our data showed that MBR-related parameters (MA, MV and MT) were not influenced by pupil dilation, we detected statistically significant changes for all variables derived from the pulse-waveform analysis. Pupil size is a major determinant of the image quality obtained with the LSFG instrument.[28] As the relationship between pupil size and MBR or the pulse-waveform analysis is as yet unexplored, pupil size cannot be ruled out as a causative factor. As a further factor worth discussing, a muscarinic antagonist (tropicamide) rather than an alpha-adrenergic agonist (e.g. phenylephrine) was used for pupil dilation as the latter has been reported to alter ocular perfusion in some studies.[49] Nevertheless, the possibility that topical tropicamide may in like manner influence ocular circulation cannot be excluded. After all, it should be considered that the differences observed between measurements with and without pupil dilation were marginal in absolute terms (Table 3).

In recent years, various LSFG-derived parameters have been purported to hold the potential of serving as biomarkers for age-related changes in ocular perfusion.[32–34,36,40] The present study demonstrated statistically significant relationships between the vast majority of LSFG-derived parameters and age. Parameters that statistically significantly decreased with age were MA, MV, MT, blowout time (BOT), blowout score (BOS) and flow acceleration index (FAI). In contrast, skew, acceleration time index (ATI), falling rate (FR) and resistivity index (RI) showed a statistically significant positive correlation with age. The pulse-waveform variables FR (r = 0.747) and BOT (r = -0.714) were most strongly correlated with age. The buffering capacity of the large arteries (i.e. the Windkessel effect) diminishes with age causing an increased systolic pulse pressure as well as a more pulsatile blood flow pattern with lower diastolic flow rates.[50] In the present study, these effects were reflected by the observed age-related increase in FR, indicating a more sudden drop-off in blood flow after the peak, as well as the decrease in BOT with age, expressing diminished peripheral perfusion during the diastole. Our findings are in accordance with previous studies of Japanese populations that reported a similar age-dependency of BOT [32–34,40] and FR.[34,40]

In the present study, we extracted pulse-waveform variables from the ONH microvasculature only. It must be noted that ONH microvasculature was defined as the ONH area free from visible vessels on the LSFG image as determined by a predefined threshold. Hence, it cannot be excluded that the analyzed area not only comprised ONH capillaries but also larger subsurface vessels. Moreover, due to the two-dimensional principle of LSFG, the technique does not permit depth-resolved measurements of perfusion. Thus, the relative attribution of the different ONH depth layers to the obtained absolute LSFG signal is unclear.

In addition, the question remains as to whether the age-related perfusion alterations detected with LSFG in the ONH microvasculature are attributable to local (ocular) or systemic (cardiovascular) changes or both. We believe that this question cannot be addressed without simultaneous measurements of the actual arterial pulse waveform, which are difficult to perform non-invasively. As a surrogate, we used pulse pressure amplitude (PPA) in this study and generally detected weaker correlations of the pulse-waveform analysis derived parameters with PPA than with age. These findings are in agreement with Tsuda et al.[34], who found weaker correlations of BOT with PPA (r = -0.44, p<0.01) than with age (r = -0.68, p<0.0001). The same held for the index FR, which was reported to be in stronger correlation with age (r = 0.61, p<0.0001) than with PPA (r = 0.38, p<0.01). To conclude, we acknowledge the fact that the LSFG-based pulse-waveform analysis of the ONH microvasculature requires further validation (e.g. by direct comparison with invasive measurements of the arterial pulse waveform). Even though we found a strong age-dependence of several LSFG parameters, however, the contribution of ocular and/or systemic age-related vascular changes cannot be clearly distinguished.

To date, the body of evidence on gender differences in ocular blood flow is markedly limited.[51] One recent report by Yanagida et al. was the first to explore sex-related differences in the ONH blood flow in a sample of 103 healthy Japanese subjects.[40] Using LSFG, they reported significantly higher MA, RR, FAI, ATI and RI in female subjects and significantly higher BOS in the male subgroup. In our study, however, of all investigated LSFG-derived parameters, only ATI and RR demonstrated a sex-related difference with statistically significantly higher mean values in females. Nevertheless, it needs to be stated that the current study was not primarily designed to detect gender differences. Furthermore, our data showed only a moderate level of repeatability for RR and, hence, this parameter must be interpreted with care.

Limitation to this work may be found. First and foremost, this study only included subjects without significant lens opacities (LOCS-II grade ≤ 2), nevertheless, it cannot be excluded that minor opacifications of the crystalline lens inherent to aging might have caused an underestimation of MBR in older subjects.[36] Furthermore, the framework of this study did not include genotyping of the cohort. Moreover, with respect to the evaluation of measurement repeatability, only three consecutive measurements before and after pupil dilation were analyzed. In addition, it is not denied that the study might have been underpowered to detect subtle sex-related differences in ONH perfusion as the sample size calculation was performed based on a single linear correlation analysis between MA and age.

To conclude, the present study demonstrated the feasibility of LSFG to quantify ocular blood flow in white subjects. We reported excellent repeatability for the majority of LSFG-based parameters of perfusion at the ONH. Pupil dilation significantly improved the measurement success rate but had no effect on repeatability or absolute MBR values. In addition, our data indicated that especially the pulse-waveform variables FR and BOT may hold the potential of serving as biomarkers for age-related changes in the ocular perfusion profile. Due to its non-invasiveness, examiner and patient friendliness and its real-time nature, LSFG represents a promising method to expand the body of evidence on sex-related differences in ocular blood flow in future studies with appropriate sample size.

## Supporting Information

S1 Table Raw data. This file contains all data analyzed in the present study.
(XLSX)
